# The Pre-/Post-Transplant Hepatitis C Antibody Associated with the IL-28B RS8099917 TT Genotype and miRNA-122 Expression May Protect Acute Cellular Rejection After LDLT

**DOI:** 10.3390/cimb46110760

**Published:** 2024-11-10

**Authors:** King-Wah Chiu, Yu-Cheng Lin, Wei-Feng Li, Kuang-Tzu Huang, Li-Wen Hsu, Chih-Chi Wang

**Affiliations:** 1 Division of Hepato-Gastroenterology, Department of Internal Medicine, Kaohsiung Chang Gung Memorial Hospital, Kaohsiung 83301, Taiwan; c471026@ms6.hinet.net; 2 Liver Transplantation Program, Kaohsiung Chang Gung Memorial Hospital, Kaohsiung 83301, Taiwan; dr.linyucheng@gmail.com (Y.-C.L.); webphone0613@yahoo.com.tw (W.-F.L.); 3 College of Medicine, Chang Gung University, Tao-Yuen 33302, Taiwan; 4 Department of Surgery, Kaohsiung Chang Gung Memorial Hospital, Kaohsiung 83301, Taiwan; 5 Institute for Translational Research in Biomedicine, Kaohsiung Chang Gung Memorial Hospital, Kaohsiung 83301,Taiwan; huangkt@cgmh.org.tw (K.-T.H.); hsuliwen@ms55.hinet.net (L.-W.H.)

**Keywords:** acute cellular rejection, hepatitis C virus, IL-28B, liver transplantation, microRNA-122, single-nucleotide polymorphism

## Abstract

This study aimed to investigate the relationship between the IL-28B SNP rs8099917 genotype, miRNA-122 expression, and the immune mechanism of ACR after LT using anti-HCV antibody calibration. A total of 45 patients with HCV received LT. IL-28B SNP rs8099917 genotyping was used to divide patients into TT and GT groups. The relative expression levels of miRNA-122 were calculated by quantitative PCR. Anti-HCV titers before and after LT were tracked to observe the relationship with ACR. The ACR rates were 27.6% for genotype TT and 62.5% for genotype GT, indicating a significantly higher rate in the GT group compared to the TT group (*p* = 0.024). In the rs8099917 genotype, TT was significantly associated with higher serum miRNA-122 levels than GT (*p* < 0.001). The TT group had significantly better outcomes than the GT group (*p* = 0.005). The Mann–Whitney U test showed significant differences in pre-LT and post-LT anti-HCV titers between the IL-28B genotypes (TT and GT) (*p* values of 0.006 and 0.027, respectively). These results suggested that the IL-28B rs8099917 genotype TT may play a significant role in modulating immune responses, both in terms of anti-HCV titers and the risk of ACR, possibly mediated through miRNA-122 levels.

## 1. Introduction

The immune mechanism of hepatitis C virus (HCV) infection is indeed complex and involves multiple interactions between host and viral factors [1]. When a patient is infected with HCV, the immune system produces antibodies against HCV, but these antibodies are not protective and cannot eliminate the virus from the body. Non-protective antibodies do not provide immune protection against HCV, possibly due to the high mutation rate of HCV, which makes it difficult for antibodies to effectively neutralize the virus [2,3]. These antibodies can only be used as indicators for diagnosing HCV infection. Anti-HCV antibodies can be used for clinical diagnosis to determine whether a person has been infected or is currently infected with HCV. As we know, HCV can evade the immune system by impairing the signaling of immune cell receptors and affecting the function and survival of both innate and adaptive immune cells. The host immune response to HCV includes both innate and adaptive immunity. Innate immunity involves interferons and natural killer cells, while adaptive immunity involves CD4+ and CD8+ T cells [4]. The immune response mechanism of HCV infection activates an innate immune response, including the production of interferons, which can inhibit viral replication [5,6]. Additionally, host cytotoxic T lymphocytes and natural killer cells play important roles in the process of viral clearance. However, the HCV uses various strategies to evade the host immune response, including avoiding the effects of neutralizing antibodies and spreading through cells [7,8]. Although anti-HCV antibodies are not protective, they represent the immune response of the host immune system to HCV infection. Therefore, exploring HCV antibody titers is valuable [9]. While anti-HCV is not a protective antibody against HCV, its development appears to be an immune response in which T cells are responsible for attacking the virus and signaling natural killer cells and interferons to protect the host. Acute cellular rejection (ACR) remains a significant problem after liver transplantation (LT). It represents a genetic pathological response of the immune system to the potential effects of immunosuppressants, particularly in HCV-associated LT [10]. Liver grafts may face an immune response from the host and a residual viral biological attack. The IL-28B single-nucleotide polymorphism (SNP) rs8099917 is known to help fight and spontaneously clear HCV infection due to its genetic characteristics [11]. Additionally, the host’s own small non-coding RNAs, including miRNA-122, miRNA-301, miRNA-133a, and miRNA-21, may be expressed in the liver with varying degrees of genetic upregulation and downregulation under different pathogenic conditions [12]. In particular, miRNA-122 is indeed a significant player in the context of ACR after LT [13]. Meanwhile, the host factors, such as genetic polymorphisms, can influence the outcome of HCV infection and the response to treatment. Studies have shown that the rs8099917 genotype may influence the outcomes of liver transplantation, including the risk of ACR. However, the exact mechanisms and the extent of this influence are still under investigation [14,15]. This study aims to explore the relationship between the IL-28B SNP rs8099917 genotype, miRNA-122 expression, and the pathological mechanism of anti-HCV levels on ACR after LT.

## 2. Materials and Methods

### 2.1. Patients

A total of 45 anti-HCV-positive patients receiving LDLT were included in this study, comprising 29 males and 16 females, with a mean age of 58.16 ± 7.02 years, of whom 42.2% had hepatocellular carcinoma. The inclusion criteria required anti-HCV-positive patients receiving LDLT who experienced a sudden onset of alanine aminotransferase (ALT) flare-up greater than twice the normal limit (U/L), who experienced acute jaundice with total serum bilirubin greater than 1.4 mg/dL, and who underwent percutaneous liver biopsy for histopathologic diagnosis. The exclusion criteria included patients with negative serum anti-HCV antibodies, positive serum HBsAg levels, underlying psychiatric conditions, primary biliary cirrhosis, alcohol-related liver disease, or a history of pediatric LT. The complete clinical patient profile is shown in Table 1. According to IL-28B SNP rs8099917 genotyping, the patients were divided into two groups: 29 cases with the TT genotype and 16 cases with the GT genotype. All patients received interferon plus ribavirin or direct-acting antiviral agents, including Harvoni (i.e., 400 mg of sofosbuvir plus 90 mg of ledipasvir), or a combination of 400 mg of sofosbuvir/60 mg of daclatasvir/800 mg of ribavirin for HCV infection whenever possible. All recipients received the same immunosuppression protocol, being administered mycophenolate mofetil at 250 mg of Q12H PO and tacrolimus at 1 mg of QD PO, with doses adjusted according to mycophenolic acid plasma levels > 3.0 μg/mL and tacrolimus blood concentrations of 5–10 ng/mL. Our exclusion criteria included pediatric transplantation, deceased donor liver transplantation, HBsAg-positive patients, primary biliary cholangitis, biliary atresia, Wilson’s disease, polycystic disease, and re-transplantation.

### 2.2. Anti-HCV Antibody

Serum samples for anti-HCV were collected on the day before LT and at the same time as liver biopsy when clinically necessary after LT. Anti-HCV was detected using the Cobas e411 analyzer through an automated electrochemiluminescence immunoassay, executed with the Elecsys Anti-HCV II assay kit (Roche Diagnostics GmbH, Mannheim, Germany) [16].

### 2.3. IL-28B Single-Nucleotide Polymorphism

Genomic DNA was extracted from the peripheral blood mononuclear cells of recipients on the pre-operative day using the QIAamp DNA Blood Mini Kit (Qiagen, Hilden, Germany). IL-28B SNP rs8099917 genotypes were studied using a 7500 Fast Real-Time PCR System (Applied Biosystems, Foster City, CA, USA) with Custom TaqMan SNP Genotyping Assays (Applied Biosystems) for allele discrimination. The real-time PCR reactions were performed in 96-well microplates, using the ABI 7500 Fast Real-Time Polymerase Chain Reaction System (Applied Biosystems International, Framingham, MA, USA) in accordance with the manufacturer’s instructions. The IL-28B SNP rs8099917 was defined as TT, GT, or GG genotype, as recommended by the manufacturer. All genotypes of IL-28B SNP rs8099917 were assayed in duplicate to assess inter-assay precision [17,18]. Only two genotypes, TT and GT, were detected in this study.

### 2.4. MicroRNA-122

Samples of miRNA-122 were collected at the same time as liver biopsy when clinically necessary after LT. miRNA-122 levels were extracted from the plasma using the miRNeasy Mini Kit (Qiagen 217004, Beijing, China) according to the manufacturer’s protocol. Reverse transcription (RT) was performed with 1 µg of RNA using the First-Strand cDNA Synthesis Kit (Promega, Madison, WI, USA) or miScript RT Kit (Qiagen, Hilden, Germany) for the transcription of miRNA according to the manufacturer’s instructions. Using the ABI TaqMan Fast Universal PCR master mix or TaqMan Universal PCR master mix for miRNA (Applied Biosystems, Foster City, CA, USA), we performed RT-PCR on an ABI 7500 Fast Real-Time PCR System with the SDS 1.4 program. The cycle threshold (Ct value), which was inversely correlated with miRNA level, was defined as the number of cycles required for the fluorescent signal to cross the threshold in quantitative PCR. Comparative RT-PCR data, including non-template controls, were obtained in triplicate. Due to the very low concentration of miRNA-122 in blood, it was crucial to select the appropriate reference gene and use sensitive detection methods. One of these methods was to use U6 snRNA as the reference gene, which was corrected and relatively stable in this study. The fold increase in cytokine mRNA expression was calculated using the comparative 2^−ΔΔCt^ method, where Ct represents the threshold cycle for each transcript. The expression of the miRNA was defined based on Ct, and relative expression levels were calculated as 2^^^[(Ct of miR−122) − (Ct of U6)] after normalization with reference to the expression of small nuclear RNA U6 [19].

### 2.5. Liver Biopsy Interpretation

All suspected clinical organ rejections after LT were assessed by the first author of this study. The histopathological diagnostic criteria for all pathological graft rejections were defined based on the 1995 Banff classification, and the severity grades were characterized by the rejection activity index [20].

### 2.6. Institutional Review Board Statement

The study protocol was approved and authorized by the ethics committee of our hospital (approval number: 202300159B0) and performed in accordance with the ethical principles of the Declaration of Helsinki.

### 2.7. Informed Consent Statement

Each participant provided written informed consent before participating in this study.

### 2.8. Statistics

Statistical analyses were performed using SPSS (version 22.0; SPSS Inc., Chicago, IL, USA). Descriptive values were expressed as mean ± standard deviation and percentages. Categorical variables were compared using the Mann–Whitney U test, the chi-squared test, or Fisher’s exact test, and continuous variables were compared using Student’s *t*-test. All tests were two-tailed, and a *p*-value of <0.05 was considered statistically significant. Log Rank (Mantel-Cox) and Kaplan–Meier curves were used for outcome analysis. Multivariate analysis was used to correlate the relationship between the co-factors.

## 3. Results

Although the IL-28B SNP rs8099917 can be defined as three genotypes (TT, GT, or GG), only two genotypes (TT and GT) were detected in this study. Therefore, the subjects were divided into TT and GT groups for further discussion.

### 3.1. The Relationship Between miRNA-122 Expression and IL-28B rs8099917

**Figure 1:**
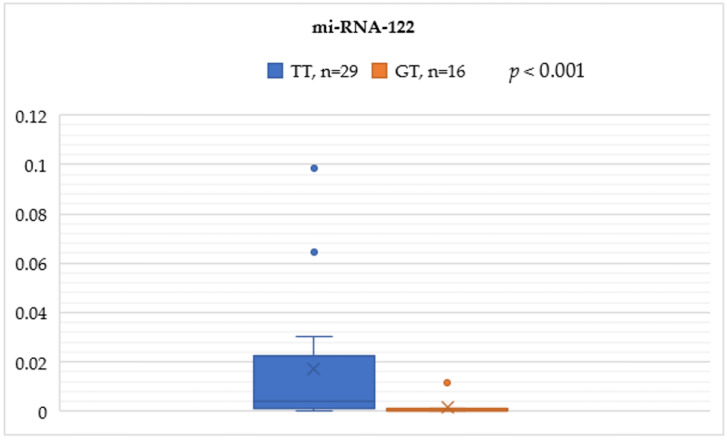
Mann–Whitney test analysis for the miRNA-122 expression comparisons between the IL-28B SNP rs8099917 genotypes TT and GT.

In the rs8099917 genotype, the TT group was significantly associated with higher serum miRNA-122 levels compared to the GT group (0.0170 ± 0.0283 vs. 0.0016 ± 0.0038, *p* < 0.001) (Figure 1).

### 3.2. The Relationship Between Acute Cellular Rejection and IL-28B rs8099917

The ACR rates were 27.6% (8/29) for the TT genotype and 62.5% (10/16) for the GT genotype, indicating a significantly higher rate in the GT group compared to the TT group (*p* = 0.024) (Table 2).

**Figure 2:**
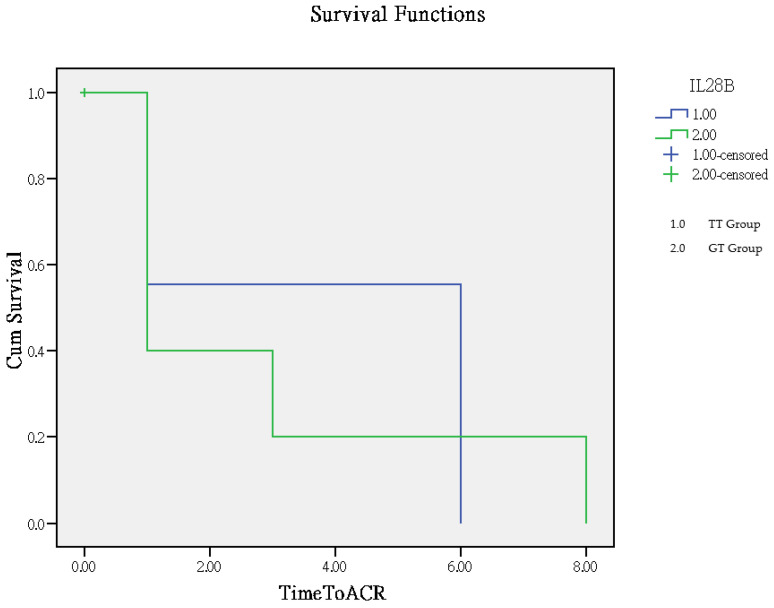
Analysis of clinical outcomes using the Kaplan–Meier curve for time to rejection.

The time to rejection ranged from 1 to 8 months, with a mean of 2.8 months in the GT group and 3.9 months in the TT group. The TT group had significantly better outcomes compared to the GT group (*p* = 0.005), as determined by Log Rank (Mantel–Cox) overall comparisons, which are illustrated in the Kaplan–Meier curve (Figure 2).

### 3.3. The Relationship Between Anti-HCV Titer and IL-28B rs8099917

The mean rank and rank sum of pre-LT anti-HCV and post-LT anti-HCV showed that the IL-28B genotype TT group had higher values for both variables. The Mann–Whitney U test indicated a significant difference in pre-LT anti-HCV and post-LT anti-HCV between the different IL-28B genotypes (TT and GT) (*p* values of 0.006 and 0.027, respectively), suggesting that the IL-28B genotype significantly affects these variables. A negative Z-score indicates a higher rank sum in the group with the IL-28B genotype TT. These results suggest that the IL-28B genotype has a significant effect on pre-LT and post-LT anti-HCV titers, with the TT group showing higher values for these variables (Table 3A,B).

Multivariate analysis with 95% confidence intervals showed the correlation between IL-28B rs8099917 genotyping, pre-LT/post-LT anti-HCV titers, and ACR (Table 4 and Table 5). Table 4 shows the effects of different variables on multiple dependent variables (miRNA-122, pre-LT anti-HCV, post-LT anti-HCV, ACR).

#### 3.3.1. Pre-LT Anti-HCV Titer

- Intercept: B = 29.173, indicating a predicted value of 29.173 for pre-LT anti-HCV when all other variables are zero, which is a highly significant result (*p* < 0.001).
- IL-28B TT: B = 23.559 (*p* = 0.002, 95% CI: 9.111 to 38.006, observed power = 0.895), indicating that the predicted value of pre-LT anti-HCV increased by 23.559 when IL-28B was the TT genotype, which is significant (*p* = 0.002).

#### 3.3.2. Post-LT Anti-HCV Titer

- Intercept: B = 50.304, indicating a predicted value of 50.304 for post-LT anti-HCV when all other variables are zero, which is a highly significant result (*p* < 0.001).
- IL-28B TT: B = 29.082 (*p* = 0.039, 95% CI: 1.593 to 56.572, observed power = 0.550), indicating that the predicted value of post-LT anti-HCV increased by 29.082 when IL-28B was the TT genotype, which is significant (*p* = 0.039).

#### 3.3.3. miRNA-122

- Intercept: B = 0.002, indicating a predicted value of 0.002 for miRNA-122 when all other variables are zero. This result is not significant (*p* = 0.778).
- IL-28B TT: B = 0.015 (*p* = 0.038, 95% CI: 0.001 to 0.030, observed power = 0.554), indicating that the predicted value of miRNA-122 increases by 0.015 when IL-28B is the TT genotype, which is significant (*p* = 0.038).

#### 3.3.4. ACR

- Intercept: B = 0.625, indicating a predicted value of 0.625 for ACR when all other variables are zero, which is a highly significant result (*p* < 0.001).
- IL-28B TT: B = −0.349 (*p* = 0.022, 95% CI: −0.is to −0.053, observed power = 0.643), indicating that the predicted value of ACR was reduced by 0.349 when IL-28B is the TT genotype, which is significant (*p* = 0.022).

For the IL-28B TT genotype, the parameter estimate (B) is −0.349, with a standard error of 0.147. The t-value is −2.380, and the *p*-value (Sig.) is 0.022, which is less than 0.05, indicating that this result is statistically significant. The 95% confidence interval for this parameter ranges from −0.645 to −0.053. This means that individuals with the IL-28B TT genotype have an ACR that is, on average, 0.349 units lower than those with the IL-28B GT genotype. The negative B value suggests a negative association between the IL-28B TT genotype and ACR. The observed power for this parameter is 0.643, indicating a moderate level of statistical power. In summary, the IL-28B TT genotype is significantly associated with a lower ACR compared to the IL-28B GT genotype.

In conclusion, IL-28B has a significant effect on all dependent variables. Specifically, when the SNP rs8099917 is the TT genotype, the values of miRNA-122, pre-LT anti-HCV, and post-LT anti-HCV increase, while the value of ACR decreases. The intercept term is significant in all models, suggesting that the base level of the dependent variables is important even after controlling for other variables. These results suggest that the IL-28B rs8099917 genotype has a significant effect on these biomarkers, which may be of great significance for related medical research and clinical applications.

## 4. Discussion

In the context of LT, the immune mechanism of HCV infection is extremely complex, involving interactions between the host, the new liver graft, and viral agents. This study aims to explore the roles of IL-28B and microRNA, which have shown positive effects on HCV, in addressing the challenge posed by the lack of an effective vaccine. Indeed, serum HCV antibodies are not protective and cannot eliminate the virus from the body. Based on our current results, anti-HCV titers may signal an association with acute cellular rejection (ACR) after LT. Specifically, lower pre-LT anti-HCV titers and higher post-LT anti-HCV titers may enhance the immune response to liver grafts. The immune response mechanism of HCV infection activates an innate immune response, including the production of interferons. Host cytotoxic T lymphocytes and natural killer cells play important roles in the process of viral clearance. Meanwhile, the host’s immune system also faces the new liver graft and expresses miRNA-122 for upregulation, leading to the avoidance of ACR after LT. Although anti-HCV antibodies are not protective, they represent the immune response of the host immune system to HCV infection. Therefore, exploring HCV antibody titers is valuable [9]. While anti-HCV is not a protective antibody against HCV, its development appears to be an immune response in which T cells are responsible for attacking the virus and signaling natural killer cells and interferons to protect the host. Regarding the outcome of LT, the anti-HCV response to host immunity seems to not only improve HCV treatment control but also protect against ACR in LT situations, as evidenced by our current results. Since anti-HCV is not a protective antibody, a transplanted organ is perceived as a foreign organ. Even in patients with the TT genotype and sustained virologic response (SVR), there is an immune response that enhances the body’s immunoglobulin, leading to a relative increase in anti-HCV titers after LT.

It is well known that the IL-28B SNP rs8099917 genotype benefits HCV clearance, whether spontaneously or through PEG interferon administration [21,22]. In our current study, it is suggested that the rs8099917 GT genotype may be significantly associated with ACR after LT compared to the TT genotype. The IL-28B genotype has been studied in the context of LT, and research indicates that this genotype can influence the immune response and outcomes post-LT. The TT genotype is associated with a different immune response compared to other genotypes [21]. The rs8099917 SNP of the IL-28B gene significantly affects the regulation of the immune system. IL-28B belongs to the interferon-lambda (IFN-λ) family, and these polymorphisms affect its expression levels, which in turn modulate the immune response. Studies have shown that individuals carrying the rs8099917 TG/GG genotype have a significant increase in seroconversion after influenza vaccination. Individuals with these genotypes had increased IL-4 production and HLA-DR expression in T cells and B cells after vaccination. In addition, IL-28B can promote the production of Th1 cytokines and IFN-γ while inhibiting the production of Th2 cytokines, such as IL-4, IL-5, and IL-13, thereby regulating the balance of immune responses. Overall, IL-28B rs8099917 SNPs play an important role in the regulation of the immune system by influencing Th1/Th2 homeostasis and B-cell responses [21,22]. The frequency of IL28B rs8099917 genotypes can vary significantly depending on the population and ethnicity. Studies from Uruguay show that the distribution of IL28B rs8099917 genotypes in the general population was 60.9% TT, 33.7% TG, and 5.4% GG [23]. This distribution is similar to findings from other populations. In our previous study, the genotyping of IL28B rs8099917 showed that 41 (82%) were TT, 9 (18%) were GT, and 0 were GG on post-transplant day 30 (KW Chiu, et al. PLoS One, 2016, 11, e0156846). These results are consistent with the findings of our current study.

In our recent report, different clinicopathological situations express different miRNAs with varying upregulation and downregulation effects in the context of LT [24,25]. Specifically, miRNA-122 has a close correlation with ACR in LT patients. The expression of liver miRNAs is significantly higher compared to serum miRNAs. When organ tissues are not available, serum miRNAs can be a simple method to aid in the diagnosis of acute jaundice after LT. miRNA-122 plays an important role in organ immunity and rejection. In terms of immunomodulation, miRNA-122 is predominantly expressed in the liver and plays a crucial role in liver disease and LT. It can modulate the immune response and affect the liver’s immune tolerance. Studies have shown that miRNA-122 can influence the activity and function of immune cells by regulating various immune-related genes. Regarding the rejection of transplanted organs, miRNA-122 is considered a potential biomarker for predicting and diagnosing liver transplant rejection in LT. Studies have found that the expression level of miRNA-122 is associated with rejection after LT, with low expression levels potentially predicting the occurrence of rejection. Overall, miRNA-122 significantly impacts organ immunity and rejection by modulating immune responses and serving as a biomarker for rejection. Recent reports have shown that lower levels of miRNA-122 are associated with an increased risk of rejection. Hence, monitoring miRNA-122 levels could potentially serve as a biomarker to predict or diagnose ACR, allowing for earlier intervention and management [25]. Based on our previous studies, the IL-28B SNP genotype and miR-122 levels may differ between donor liver tissue and recipient blood. This discrepancy can be an interesting topic to explore. We propose a so-called homogeneous phenomenon when there are different genotypes between the donor and the recipient. When the genotype does not match the recipient’s immune response and histopathological rejection conditions, it may affect the stability of the liver graft [9]. Regarding the outcome of LT, the anti-HCV response to host immunity appears to not only improve HCV treatment control but also protect against ACR in LT situations. Based on our current results, the IL-28B TT genotype is associated with higher anti-HCV titers both before and after LT compared to the GT genotype. This could indicate a stronger immune response in individuals with the TT genotype. Higher miRNA-122 levels might be linked to better regulation of immune responses, potentially reducing the risk of acute cellular rejection. Our study suggests that the IL-28B TT genotype may play a significant role in modulating immune responses, both in terms of anti-HCV titers and the risk of acute cellular rejection, possibly mediated through miRNA-122 levels. The IL28B genotype, particularly the rs8099917 TT genotype, is associated with a better immune response and better treatment outcomes in chronic HCV infection. It may provide a positive response on the immune system against HCV. However, its direct impact on specifically enhancing the immune response to ACR after LT is still under investigation. Our current results may provide new scientific insights into the genetic regulation of IL28B and miRNA-122 and their roles in the mechanism of ACR.

Our study has some limitations. First, the sample size is relatively small, and the study is single-centered. Nevertheless, we consider the risk of bias to be very small due to the standardization of pre-LT clinical and laboratory assessments, sophisticated LT surgical techniques, and post-LT protocols for immunosuppression regimens and graft outcome surveillance. Second, we could determine the association between changes in anti-HCV antibody titers before and after LT procedures and allograft injury only through a retrospective review of medical records. There were a lack of data regarding the immunosuppressive treatment schedule and the serum levels of immunosuppressive drugs to enable comparisons between anti-HCV antibody titers. Future prospective studies may be warranted to both identify the role of molecular expression levels in allograft rejection and clarify the mechanisms underlying the association between DAA-induced anti-HCV antibody titer fluctuations and immune reactions to post-LT allograft injury. The findings of such studies may shed light on measures for optimizing pre-LT plans and donor selection, as well as serve as a reference for modifying anti-HCV treatment protocols for potential liver allograft recipients to reduce the incidence and severity of ACR. In particular, a simple Student *t*-test interpretation was statistically significant in the analysis of IL-28B TT and miRNA-122. Nonetheless, we judged the risk of bias to be very small, as the standardization of pre-LT clinical and laboratory assessments, the monitoring of immunosuppressive regimens and graft outcomes after LT, the use of multivariate analyses to examine the strength of statistical data to confirm their reliable associations, and increased study funding with a view to collecting more cases may be more convincing. Moreover, immune response to an implanted graft may not be same as that of the HCV virus. Besides the small sample size, the major drawback of this study is not taking into consideration the genetic backgrounds of donors, as the outcome of liver transplant critically depends on both recipient and donor genetic makeups.

In conclusion, IL-28B has a significant effect, especially when the SNP rs8099917 is the TT genotype. The current results suggest that the IL-28B rs8099917 genotype has a significant effect on these biomarkers, which may be of great significance for related medical research and clinical applications.

## Figures and Tables

**Table 1:** The clinical profiles of anti-HCV-positive recipients with different IL-28B SNP rs8099917 TT and GT genotypes underwent living donor liver transplantation.

Category	TT Group No. (%)	GT Group No. (%)	*p* Value
Cases	29 (64.4)	16 (35.6)	
Gender, M/F	17/12	12/4	NS
Age (mean ± SD)	57.41 ± 6.65	59.94 ± 7.84	NS
Combined with HCC	25 (86.2)	10 (62.5)	NS
HCV genotype (1a/1b/2/undetected)	2/12/5/10	0/4/5/7	
*Pre-LT*			
AST	39.69 ± 16.77	63.81 ± 57.88	<0.05
ALT	33.83 ± 21.17	67.94 ± 108.88	NS
Bil. T	1.40 ± 0.72	2.03 ± 1.15	<0.05
HCV RNA	11 (37.9)	6 (37.5)	NS
Anti-HCV titer	52.73 ± 26.52	29.17 ± 14.29	<0.005
DAA treatment	6 (20.7)	5 (31.3)	NS
*Post-LT*			
AST	35.41 ± 22.55	64.13 ± 41.17	<0.005
ALT	42.62 ± 39.00	73.13 ± 45.98	<0.05
Bil. T	0.64 ± 0.28	0.69 ± 0.37	NS
HCV RNA	6 (20.7)	2 (12.5)	NS
Anti-HCV titer	79.39 ± 45.56	50.30 ± 40.21	<0.05
DAA treatment	10 (34.5)	6 (37.5)	NS

Alb: albumin; ALT: alanine aminotransferase; AST: aspartate aminotransferase; Bil. T: total bilirubin; HCC: hepatocellular carcinoma; LT: liver transplantation.

**Table 2:** The relationship between acute cellular rejection and IL-28B SNP rs8099917 in patients with HCV relative living donor liver transplantation.

IL-28B SNP rs8099917	Acute Cellular Rejection N (%)	*p* Value
Genotype TT, *n* = 29	8 (27.6)	0.024
Genotype GT, *n* = 16	10 (62.5)	

HCV: hepatitis C virus; IL: interleukin; SNP: single-nucleotide polymorphism. Mann–Whitney U test (2-tailed); <0.05 indicates a significant difference.

**Table 3:** (**A**) The Mann–Whitney U test for analyzing the relationship between pre-/post-LT anti-HCV titer and IL-28B TT and GT genotypes. (**B**) Significant difference between the pre-/post-LT anti-HCV titer and IL-28B TT and GT genotypes.

**(A)**				
**Ranks**	**IL28B**	**N**	**Mean Rank**	**Sum of Ranks**
Pre-LT anti-HCV	TT	29	26.97	782.00
	GT	16	15.81	253.00
	Total	45		
Post-LT anti-HCV	TT	29	26.21	760.00
	GT	16	17.19	275.00
	Total	45		
**(B)**				
**Test Statistics (a)**	**Pre-LT Anti-HCV**		**Post-LT Anti-HCV**	
Mann–Whitney U	117.000		139.000	
Wilcoxon W	253.000		275.000	
Z	−2.729		−2.207	
Asymp. Sig. (two-tailed)	0.006		0.027	

**(a)**, grouping variable: IL28B.

**Table 4:** Multivariate tests ^c^ for IL-28B SNP rs8099917 and co-factors.

Effect		Value	F	Hypothesis df	Error df	Sig.	Observed Power ^a^
Intercept	Pillai’s Trace	0.811	42.847 ^b^	4.000	40.000	0.000	1.000
	Wilks’ Lambda	0.189	42.847 ^b^	4.000	40.000	0.000	1.000
	Hotelling’s Trace	4.285	42.847 ^b^	4.000	40.000	0.000	1.000
	Roy’s Largest Root	4.285	42.847 ^b^	4.000	40.000	0.000	1.000
rs8099917	Pillai’s Trace	0.037	5.074 ^b^	4.000	40.000	0.002	0.943
	Wilks’ Lambda	0.663	5.074 ^b^	4.000	40.000	0.002	0.943
	Hotelling’s Trace	0.507	5.074 ^b^	4.000	40.000	0.002	0.943
	Roy’s Largest Root	0.507	5.074 ^b^	4.000	40.000	0.002	0.943

a: Computed using alpha = 0.05; b: Exact statistic; c: Design = Intercept+IL28B.

**Table 5:** Parameter estimates for IL-28B SNP rs8099917 and co-factors.

Dependent Variable	Parameter	B	Std. Error	t	Sig.	95% Confidence Interval		Observed Power ^a^
						Lower Bound	Upper Bound	
Pre-LT anti-HCV	Intercept	29.173	5.751	5.037	0.000	17.575	40.770	0.999
	[IL-28B TT]	23.559	7.164	3.209	0.002	9.111	30.006	0.095
	[IL-28B GT]	0 ^b^	.	.	.	.	.	.
Post-LT anti-HCV	Intercept	50.304	10.943	4.597	0.000	28.236	72.372	0.994
	[IL-28B TT]	29.082	13.631	2.134	0.039	1.593	56.572	0.550
	[IL-28B GT]	0 ^b^	.	.	.	.	.	0.
miRNA-122	Intercept	0.002	0.006	0.284	0.778	−0.010	0.013	0.059
	[IL-28B TT]	0.015	0.007	2.143	0.038	0.001	0.030	0.554
	[IL-28B GT]	0 ^b^	.	.	.	.	.	.
ACR	Intercept	0.625	0.118	5.307	0.000	387	0.863	0.999
	[IL-28B TT]	−0.349	0.147	−2.380	0.022	−0.645	−0.053	0.643
	[IL-28B GT]	0^b^	.	.	.	.	.	.

a: Computed using alpha = 0.05; b: This parameter is set to zero because it is redundant. The observed power indicates the performance of the test, and the closer the value is to 1, the higher the performance of the test.

## Data Availability

The original contributions presented in this study are included in the article; further inquiries can be directed to the corresponding author.

## Abbreviations

ALF	Acute liver failure
ALT	Alanine aminotransferase
AST	Aspartate aminotransferase
HCV	Hepatitis C virus
HCC	Hepatocellular carcinoma
INR	International normalized ratio
LT	Liver transplantation
LDLT	Living-donor liver transplantation
miRNA	MicroRNA
SNP	Single-nucleotide polymorphism
